# Heart Disease in Childhood and Youth

**Published:** 1901-01

**Authors:** 


HEART DISEASE IN CHILDHOOD AND YOUTH. By CHARLES W. CHAPMAN, M. D., Durh., M. R. C. P. Lond., Physician to the National Hospital for Diseases of the Heart, Soho Square, W., etc. With an introduction by Sir Samuel Wilks, Bart., M. D., F. R. S., Physician Extraordinary to H. M. the Queen, etc. Duodecimo, pp. 101. London: Medical Publishing Co. 1900. [Price, 3 s. 6 ]

Dr. Chapman's book consists of a preliminary chapter on the causes, hygienic management and treatment of heart disease in childhood, followed by a detailed account of thirty-two selected cases. The volume is an excellent one, so good that the reviewer is tempted to quote at length from its pages. One who has any practice at all among children can not fail to be benefited by a persual of this volume, which is so clearly and carefully written that the reading of it is not a task but a pleasure. M. J. F.

